# Bringing Hope to Improve Treatment in Pancreatic Ductal Adenocarcinoma—A New Tool for Molecular Profiling of *KRAS* Mutations in Tumor and Plasma Samples

**DOI:** 10.3390/cancers16203544

**Published:** 2024-10-21

**Authors:** Ana Catarina Bravo, Bárbara Morão, André Luz, Rúben Dourado, Beatriz Oliveira, Ana Guedes, Catarina Moreira-Barbosa, Catarina Fidalgo, Luís Mascarenhas-Lemos, Maria Pia Costa-Santos, Rui Maio, Jorge Paulino, Pedro Viana Baptista, Alexandra R. Fernandes, Marília Cravo

**Affiliations:** 1 Hospital Beatriz Ângelo, 2674-514 Loures, Portugal; ana.anjos.bravo@hbeatrizangelo.pt (A.C.B.); barbara.silva.moral@ulslod.min-saude.pt (B.M.); ana.silva.guedes@ulslod.min-saude.pt (A.G.); catarina.moreira.barbosa@luzsaude.pt (C.M.-B.); catarina.fidalgo@ulslod.min-saude.pt (C.F.); rui.maio@hospitaldaluz.pt (R.M.); 2 Associate Laboratory i4HB—Institute for Health and Bioeconomy, NOVA School of Science and Technology, NOVA University Lisbon, 2829-516 Caparica, Portugal; af.luz@campus.fct.unl.pt (A.L.); r.dourado@campus.fct.unl.pt (R.D.); abb.oliveira@campus.fct.unl.pt (B.O.); pmvb@fct.unl.pt (P.V.B.); ma.fernandes@fct.unl.pt (A.R.F.); 3 UCIBIO—Applied Molecular Biosciences Unit, Department of Life Sciences, NOVA School of Science and Technology, NOVA University Lisbon, 2829-516 Caparica, Portugal; 4 Hospital da Luz Learning Health, Luz Saúde, 1500-650 Lisboa, Portugal; 5 Hospital da Luz, 1500-650 Lisboa, Portugal; luis.lemos@hospitaldaluz.pt (L.M.-L.); jorge.paulino.pereira@hospitaldaluz.pt (J.P.); 6 NOVA Medical School, 1169-056 Lisboa, Portugal; 7 Catolica Medical School, 1649-023 Lisboa, Portugal; 8 Hospital do Divino Espírito Santo, 9500-370 Ponta Delgada, Portugal; maria.pa.santos@azores.gov.pt; 9 Lisbon School of Medicine, Universidade de Lisboa, 1649-028 Lisboa, Portugal

**Keywords:** pancreatic cancer, *KRAS* mutations, liquid biopsy, ctDNA, amplification refractory mutation system, ARMS–HRMA, prognosis

## Abstract

Background/Objectives: Pancreatic ductal adenocarcinoma (PDAC) incidence is rising, and prognosis remains poor due to late diagnosis and limited effective therapies. Currently, patients are treated based on TNM staging, without molecular tumor characterization. This study aimed to validate a technique that combines the amplification refractory mutation system (ARMS) with high-resolution melting analysis (HRMA) for detecting mutations in codon 12 of KRAS in tumor and plasma, and to assess its prognostic value. Methods: Prospective study including patients with newly diagnosed PDAC with tumor and plasma samples collected before treatment. Mutations in codon 12 of KRAS (G12D, G12V, G12C, and G12R) were detected using ARMS–HRMA and compared to Sanger sequencing (SS). Univariate and multivariate analyses were used to evaluate the prognostic significance of these mutations. Results: A total of 88 patients, 93% with ECOG-PS 0–1, 57% with resectable disease. ARMS–HRMA technique showed a higher sensitivity than SS, both in tumor and plasma (77% vs. 51%; 25 vs. 0%, respectively). The most frequent mutation was G12D (n = 32, 36%), followed by G12V (n = 22, 25%). On multivariate analysis, patients with G12D and/or G12C mutations, either in tumor or plasma, had lower PFS (HR 1.792, 95% CI 1.061–3.028, *p* = 0.029; HR 2.081, 95% CI 1.014–4.272, *p* = 0.046, respectively) and lower OS (HR 1.757, 95% CI 1.013–3.049, *p* = 0.045; HR 2.229, 95% CI 1.082–4.594, *p* = 0.030, respectively). Conclusions: ARMS–HRMA is a rapid and cost-effective method for detecting *KRAS* mutations in PDAC patients, offering the potential for stratifying prognosis and guiding treatment decisions. The presence of G12D and G12C mutations in both tumor and plasma is associated with a poorer prognosis.

## Simple Summary

Pancreatic ductal adenocarcinoma (PDAC) has a rising incidence and poor prognosis due to late diagnosis and limited treatments. Currently, treatment is based solely on TNM staging, without considering molecular tumor characterization. In the present study, we validated a combined amplification refractory mutation system (ARMS) and high-resolution melting analysis (HRMA) technique for detecting mutations in codon 12 of *KRAS* in PDAC tumor and plasma samples and assessed its prognostic value. We included 88 newly diagnosed PDAC patients, treated with either surgery, chemotherapy, or best supportive treatment only. Both tumor and plasma samples were analyzed, and the most frequent mutations were G12D (36%) and G12V (25%). *KRAS* mutations G12D and/or G12C in tumors and plasma were associated with lower progression-free survival (PFS) and overall survival (OS) independently of disease stage or treatment performed. ARMS–HRMA offers a rapid, cost-effective method for detecting *KRAS* mutations and can aid in prognosis and treatment decisions.

## 1. Introduction

According to current projections, pancreatic cancer (PC) is on track to become the second leading cause of cancer-related deaths and the most fatal gastrointestinal cancer by 2030 [1,2]. Pancreatic ductal adenocarcinoma (PDAC) will account for approximately 85% of these cases and, unfortunately, the overall 5-year survival rate is only 9% across all stages [3]. This poor outcome is primarily related to a late diagnosis coupled with the absence of effective treatments. Surgical resection of the tumor, followed by 6 months of adjuvant chemotherapy (ChT), is the only potentially curative option, but less than 20% of patients have resectable disease at diagnosis [4]. For patients with borderline resectable disease, neo-adjuvant ChT is recommended [5]. These decisions are taken considering only TNM staging on the CT scan, irrespective of tumor molecular biology, which certainly accounts for tumor heterogeneity in terms of prognosis and response to medical or surgical therapies. In this way, the development of new biomarkers for early diagnosis, prediction of treatment response, and as potential targets for personalized therapies could help improve survival rates [6].

KRAS gene mutations arise early in the development of pancreatic cancer, occurring in at least 80% of PDAC cases [7,8]. More than 90% of these mutations are found in codon 12 of the KRAS protein (p.G12D, p.G12V, p.G12R, p.G12C, or p.G12A), which result from single nucleotide changes [9,10]. Despite some discrepancies, several studies have demonstrated the prognostic significance of *KRAS* mutations in both tumor tissue and liquid biopsies from PDAC patients [11]. Whereas the KRAS G12D mutation has been systematically associated with a predicted poorer overall survival [12,13,14], there have been conflicting studies regarding KRAS G12V mutation’s prognostic significance [15,16,17,18].

Additionally, the presence and quantity of circulating tumor DNA (ctDNA) containing *KRAS* mutations have been associated with worse overall survival in PDAC patients [11]. Changes to ctDNA levels during chemotherapy have been found to be better indicators of treatment response than changes in CA 19.9 levels [19,20,21,22]. Previous studies found that ctDNA levels correlated well with disease status with decreases after neo-adjuvant therapy or tumor removal, suggesting a link between ctDNA levels and tumor burden [11,22].

Besides serving as indicators of prognosis and tumor response, *KRAS* mutational status in patients with PDAC is also an opportunity for tailored treatment. Treatment with a selective inhibitor targeting KRAS G12C mutations in patients with advanced PDAC yielded promising results [23] and other inhibitors targeting KRAS, selectively or not, are also being developed [24,25,26].

Despite previous demonstrations that *KRAS* mutation subtype could influence therapeutic decisions in PDAC patients, the lack of rapid, sensitive, and cost-efficient methods for their detection has certainly limited their use in clinical practice [27]. In a recent pilot study with 30 patients, our group proposed the use of the amplification refractory mutation system (ARMS) coupled with high-resolution melting analysis (HRMA)—ARMS–HRMA—as a sensitive, specific, and straightforward method for detecting *KRAS* mutations, focusing on G12D and G12V mutations [28].

Herein, the objectives of this study were: (1) to further validate the ARMS–HRMA technique for detecting the most common four types of mutations in codon 12 of *KRAS* in tumor and plasma samples from a larger cohort of PDAC patients and (2) to evaluate its usefulness in predicting disease progression and overall mortality.

## 2. Materials and Methods

### 2.1. Study Design and Inclusion Criteria

We conducted a multicenter, prospective cohort study involving patients newly diagnosed with histologically confirmed PDAC at two referral centers: Hospital Beatriz Ângelo (Loures, Portugal) and Hospital da Luz (Lisbon, Portugal), between October 2017 and November 2022. Patients were assigned either to immediate surgery or ChT based on tumor stage and performance status (PS). For those referred to ChT, endoscopic ultrasound-guided fine-needle biopsy (EUS-FNB) was performed to confirm the tumor’s histological characteristics. During this period, 247 newly diagnosed PDAC patients were treated at both hospitals. A total of 92 PDAC patients had fresh frozen tumor available and were included in the present study. Four of them were later excluded from the final analysis because we could not extract tumor DNA for mutational analysis by ARMS–HRMA. Therefore, a total of 88 PDAC patients were included. In 17 patients, blood samples were not collected upon admission, and the final number of plasma samples analyzed were 71. A total of 30/88 patients were previously included in our previous study [28], where we only tested for G12D and G12V mutations. End of follow-up was in May 2024.

The exclusion criteria encompassed patients who had prior treatments, including surgery, chemotherapy, or radiotherapy; those referred for neoadjuvant or palliative chemotherapy with biopsies conducted at other institutions; individuals unable to undergo surgery or EUS-FNB for histologically confirmed PDAC; and patients who had direct surgery for pancreatic tumors that were not PDAC.

The study was conducted according to the guidelines of the Declaration of Helsinki and approved by the Ethics Committee of Hospital Beatriz Ângelo (1372/2015_CMOEB (approved on 31 December 2015)) and Hospital da Luz (CES/13/2018/ME (approved on 12 April 2018)), registered on the appropriate platforms and informed written consent was obtained from all participants.

### 2.2. Tumor Sample Collection

A tumor sample was obtained at diagnosis before any treatment in one of two scenarios: (1) during resection surgery for patients undergoing upfront surgery, or (2) during EUS-FNB for patients receiving neoadjuvant or palliative chemotherapy as first-line treatment. For surgical specimens, 5 mm of the tumor tissue was placed in a sterile 1 mL Eppendorf tube, kept at 4 °C, and then immediately frozen in liquid nitrogen before being stored at −80 °C for future molecular analysis. EUS was conducted under intravenous propofol anesthesia using a curved linear array echoendoscope (model GF-UCT180, from Olympus^®^, Tokyo, Japan) connected to an Aloka ultrasound machine. EUS-FNB was performed with a 22-gauge needle, with at least two passes made for diagnostic purposes. Sample adequacy was determined by either rapid on-site evaluation (ROSE) by a pathologist in the endoscopy suite or by macroscopic on-site evaluation (MOSE). Core biopsy samples were transferred into Cytolyt media and used for histological diagnosis in accordance with standard clinical practices.

### 2.3. Blood Sample Collection

Blood samples were obtained at diagnosis prior to any treatment. A small volume of blood (8 mL) was drawn into two EDTA tubes and immediately kept at 4 °C. These samples were transported to the laboratory, maintaining the 4 °C temperature, within a maximum of four hours, ideally within two. Blood components were separated by centrifugation at 2000× *g* at 4 °C and transferred into cryotubes: four containing plasma and one containing white blood cells. The cryotubes were then flash-frozen in liquid nitrogen and stored at −80 °C for future analysis.

### 2.4. KRAS Mutation Analysis

#### 2.4.1. Cell Lines

The HT-29 cell line with an epithelial morphology isolated from a primary tumor obtained from a 44-year-old white female patient with colorectal adenocarcinoma was used as a control for the wild-type (WT) *KRAS*. Other human tumor cell lines were used as controls for each *KRAS* mutation: LS174T cell line (G12D mutation), isolated from the colon of a white 58-year-old female adenocarcinoma patient with colorectal cancer; H358 cell line (G12C mutation), an epithelial-like cell that isolated from the bronchiole of a male patient with bronchioalveolar carcinoma; SW480 cell line (G12V mutation), isolated from the large intestine of a Dukes C colorectal cancer patient; PSN-1 cell line (G12R mutation), an epithelial-like morphology isolated from the pancreas of a patient with adenocarcinoma. All cell lines were obtained from the American Type Culture Collection (ATCC^®^, Manassas, VA, USA).

#### 2.4.2. Cell Culture

The HT-29 tumor cell line was cultured in Dulbecco’s Modified Eagle Medium (DMEM; Gibco™, ThermoFisher Scientific, Waltham, MA, USA). PSN-1 and H358 cell lines were grown in Roswell Park Memorial Institute Medium (RPMI; Gibco™, ThermoFisher Scientific), while the SW480 cell line was maintained in Leibovitz’s L-15 Medium (Gibco™), which includes 2 mM L-glutamine but no sodium bicarbonate. The LS174T cell line was cultured in Eagle’s Minimum Essential Medium (EMEM; Gibco™), modified to contain Earle’s Balanced Salt Solution, non-essential amino acids, 2 mM L-glutamine, 1 mM sodium pyruvate, and 1500 mg/L sodium bicarbonate. All media were supplemented with 10% (*v*/*v*) fetal bovine serum (FBS; Gibco™) and 1% (*v*/*v*) antibiotic/antimycotic (Gibco™) and maintained in 25 cm^2^ culture flasks (VWR, Radnor, Pensilvânia, EUA) at 37 °C in a 99% humidified atmosphere with 5% (*v*/*v*) CO_2_ (CO_2_ incubator, SANYO CO_2_ Incubator, Electric Biomedical Co., Osaka, Japan).

#### 2.4.3. DNA Extraction

DNA extraction from all tumor cell lines, as well as patients’ tumor and plasma samples, was carried out using the High Pure PCR Template Preparation Kit (Roche, Basel, Switzerland) following the manufacturer’s protocol, with the following modifications: for tumor samples, 30 s were added to the centrifugation times and the Elution Buffer volume was reduced to 50 μL or 30 μL for tumor samples or plasma samples, respectively. DNA quantification was performed using a Nanodrop spectrophotometer (Thermo Fisher Scientific, Waltham, MA, USA).

#### 2.4.4. Polymerase Chain Reaction (PCR)

PCR amplification was performed on extracted DNA from control cells and tumor samples to assess the presence of each respective mutation in exon 2 of the KRAS gene through Sanger sequencing (SS). The PCR mixture was comprised of 100 ng of template DNA, 0.12 μM of each primer (forward: 5′-GGT GGA GTA TTT GAT AGT GTA-3′ and reverse: 5′-TGG ACC CTGA CAT ACT CCC AAG-3′), 1 × DreamTaq™ Buffer (Thermofisher, Waltham, MA, USA), 0.8 mM of dNTPs Mix (NzyTech, Lisbon, Portugal), 0.15 units DreamTaq™ (NzyTech, Lisbon, Portugal), resulting in a final volume of 20 μL. The reactions were performed on a DNA Engine^®^ Thermal Cycler (Bio-Rad, Hercules, CA, USA) using the following program: an initial denaturation step for 5 min at 95 °C, followed by 30 cycles of 95 °C for 30 s, 61 °C for 30 s for tumor samples or 53 °C for 30 s for plasma samples, and 72 °C for 20 s. KRAS exon 2 amplification products from tumor samples were sequenced in STABVIDA (Setubal, Portugal). The chromatograms were analyzed using the FinchTV software (Geospiza, Inc., Seattle, WA, USA, version 1.4.0).

#### 2.4.5. ARMS–HRMA

Tumor and plasma samples were analyzed using the combination of the ARMS and HRMA techniques [28]. The ARMS–HRMA mixture was comprised 50 ng of template DNA (in plasma samples, 1 μL was used regardless of the concentration), 0.3 μM of a mutation-specific forward primer (G12D—5′-CTT GTG GTA GTT GGA GCT TA-3′, G12V—5′-CTT GTG GTA GTT GGA GCTTT-3′, G12R—5′-CTT GTG GTA GTT GGA GCGC-3′, G12C—5′-CTT GTG GTA GTT GGA GCGT-3′) and 0.3 μM of a common reverse primer (5′-CTC TAT TGT TGG ATC ATA TTCG-3′), 2% (*v*/*v*) of DMSO (Merck KGaA, Darmstadt, Germany), and 1x of Supreme NZYTaq II Green Master Mix (NzyTech, Lisbon, Portugal), resulting in a final volume of 10 μL. The reactions were performed in a QIAGEN Rotor-Gene Q Real-time PCR cycler 5plex (Qiagen, Hilden, Germany) using the following program: an initial denaturation step of 3 min at 95 °C, followed by 10 cycles of 30 s at 95 °C, 15 s at 54 °C for G12D mutation, 52 °C for G12V mutation, 57 °C for G12R mutation, 56 °C for G12C mutation, and then 10 s at 72 °C, followed by 25 cycles at 30 s of 95 °C, 45 s at 60 °C, and 10 s at 72 °C. The HRMA step was performed with a temperature increase from 45 °C to 90 °C, with a 90 s of pre-melt conditioning on first step and then a 1 °C increase in each step/5 s wait each step. The melting profile and derivative plot were generated and analyzed using Rotor-Gene Q Series Software 2.3.5 (Qiagen, Hilden, Germany). Each ARMS–HRMA reaction included a positive “mutant” control (gDNA from LST174T, SW480, PSN-1, and H358 cell lines for the G12D, G12V, G12R, and G12C mutations, respectively), a “wild-type” control (gDNA from the HT-29 cell line), and non-template control to rule out contamination. After subtracting the fluorescence values of the negative control, the fluorescence value at 78.5 °C (for G12D, G12V, and G12C mutations) or 79.5 °C (for G12R mutation) of every sample was normalized using the fluorescence values of the “mutant” and “WT” controls.

### 2.5. Variables and Endpoints

Baseline demographic and clinical data include factors such as gender, age, date of diagnosis, Eastern Cooperative Oncology Group Performance Status (ECOG-PS), disease stage according to the American Joint Committee on Cancer (AJCC) TNM classification, and resectability status based on National Comprehensive Cancer Network (NCCN) guidelines [29]. During follow-up, the following variables were monitored: the occurrence and timing of disease progression (whether local or metastatic), as identified through computed tomography, magnetic resonance cholangiopancreatography and/or blood analysis with CA 19.9, as well as the date of the last follow-up or death. The primary endpoint was the frequency of KRAS codon 12 mutations identified in the primary tumor and liquid biopsies of patients with PDAC using ARMS–HRMA technology and its concordance with SS. Secondary endpoints included the association of KRAS mutations in tumor and liquid biopsies with disease progression and mortality during the follow-up period.

### 2.6. Follow-Up and Treatment

Following histological confirmation of PDAC, patients were treated according to standard care practices and international guidelines. *KRAS* status in both tumor and plasma samples was assessed at a later stage, and did not impact therapeutic decisions.

### 2.7. Statistical Analysis

Categorical variables were reported as frequencies and percentages, while continuous variables were expressed as means with standard deviations or medians with interquartile ranges, depending on their distribution. The distribution of categorical variables was analyzed using the Chi-square test. For continuous variables, either the independent sample *t*-test/one-way ANOVA or the nonparametric Mann–Whitney and Kruskal–Wallis tests were applied, depending on whether the distribution was normal or non-normal, respectively, and in line with the hypothesis being tested. Rates of progression-free survival and overall survival were computed using Kaplan–Meyer estimates, along with 95% confidence intervals. Survival analysis was performed considering each mutation alone and grouping mutations in WT, G12V, and G12R vs. other mutations, considering the previously described prognosis impact. Survival curves were generated and compared using Log Rank tests. Overall survival (OS) was defined as the time from diagnosis until death from any cause. Progression-free survival (PFS) was defined as the time until disease progression, recurrence, or death. Disease progression was defined as elevation of CA 19-9 or imaging-based disease progression during follow-up (whichever occurred first). Fluctuations in CA 19-9 that were not confirmed as disease progression by imaging were not considered significant. Multivariate Cox regression was applied to adjust the prognosis associated with *KRAS* mutation status for other potential confounding factors.

## 3. Results

### 3.1. Population Baseline Characteristics

Patients’ characteristics according to tumor *KRAS* mutational status are shown in Table 1. A total of 88 patients were included, with a median age at diagnosis of 70 (IQR 63–75) years; 57% were male, 93% had ECOG-PS 0 or 1, 57% and 12% had a resectable or borderline resectable disease, respectively, and 61% were treated with surgery, with or without chemotherapy. In 71/88 patients, we had both tumor and plasma samples whereas in the remaining 17 patients, only tumor specimens were available.

Among the 88 patients included, 68 (77%) presented a mutation in codon 12 of KRAS protein by ARMS–HRMA. The most frequent mutation was G12D (36%), followed by G12V (25%) and G12R (11%). One patient had a tumor mutated for G12C mutation, and three patients carried more than one mutation. Wild-type genotypes were more frequently present in male patients (80% vs. 50%, *p* = 0.021). No other differences in patient characteristics were found between patients with wild-type and *KRAS*-mutated tumors (Table 1).

**Figure 1:**
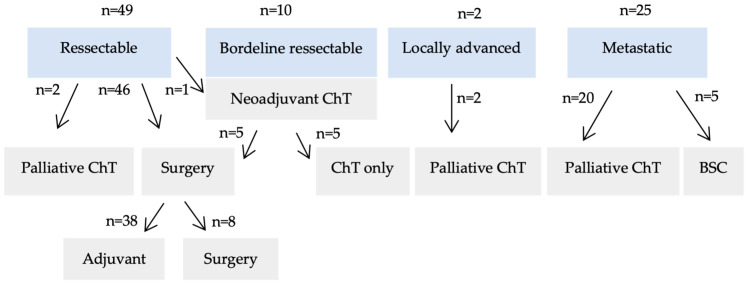
Patient management according to clinical stage. No data were available about clinical stage in n = 2 patients. BSC—best supportive care; ChT—chemotherapy.

Patient management based on clinical staging is outlined in Figure 1. No data were available about clinical stage in n = 2 patients. All 10 patients with borderline resectable disease received neoadjuvant chemotherapy (ChT) using the FOLFIRINOX regimen. Of these, five patients underwent surgery, while the other five received palliative chemotherapy due to disease progression or clinical decline. Among the 49 patients with resectable disease, 2 were deemed unfit for surgery and were offered palliative chemotherapy, while 1 received neoadjuvant ChT. First-line adjuvant ChT regimens included FOLFIRINOX-like treatments in 24 patients and gemcitabine-based regimens in 7, with the remaining patients treated with other regimens. Of the 27 patients with locally advanced or metastatic disease, 22 received palliative ChT, while the remaining 5 experienced rapid deterioration and received only best supportive care (BSC). First-line palliative ChT regimens consisted of FOLFIRINOX-like treatments for 13 patients, gemcitabine-based regimens for 5, and other regimens for the remaining 4 patients.

### 3.2. Performance of ARMS–HRMA Technique Compared to SS

#### 3.2.1. Tumor Samples

**Figure 2:**
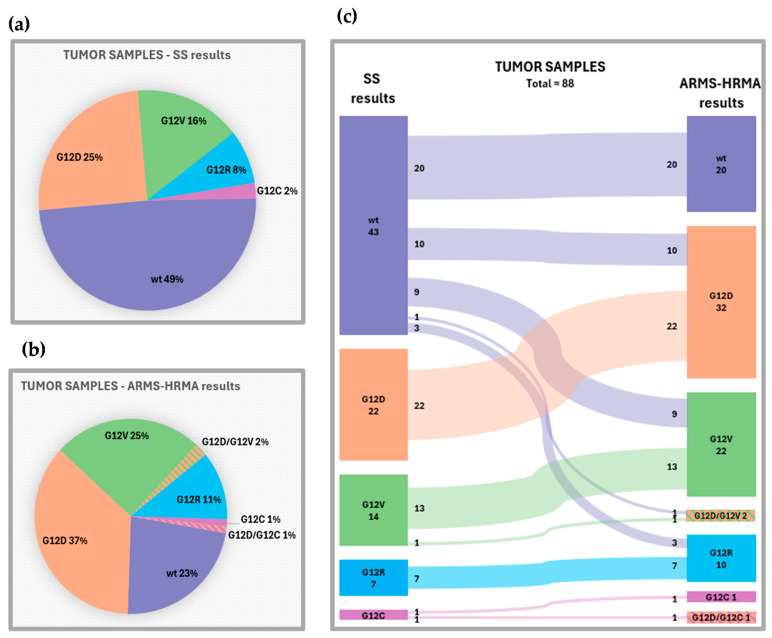
Performance of ARMS–HRMA technique compared to SS—(**a**) *KRAS* mutations detected in tumor samples, by SS, in relative frequency; (**b**) *KRAS* mutations detected in tumor samples, by ARMS–HRMA, in relative frequency; and (**c**) comparison and correspondence between both techniques, in absolute frequency.

Mutation status results were successfully obtained with SS and ARMS–HRMA from all the 88 tumor samples and are summarized in Figure 2. SS detected *KRAS* mutations in 51.1% of tumor samples (n = 45): G12D in 22, G12V in 14, G12R in 7, and G12C in 2 patients. ARMS–HRMA was able to detect mutations in 77.3% of tumor samples (n = 68), with total concordance with SS, except for the fact that ARMS–HRMA was able to detect two mutations in patients with only one mutation detected by SS. Thus, according to ARMS–HRMA, *KRAS* mutations in tumor samples were as follows: WT in 20, G12D in 32, G12V in 22, G12D/G12V in 2, G12R in 10, G12C in 1, and G12D/G12C in 1 patient.

#### 3.2.2. Liquid Biopsies (Plasma Samples)

Plasma samples were obtained from 71 patients, with 25.4% (n = 18) having *KRAS* mutations detected by ARMS–HRMA—G12V in 6 (8.5%), G12D in 8 (11.3%), G12R in 3 (4.2%), and G12V/G12D in 1 patient (1.4%). All mutations found in plasma agreed with mutations found in primary tumors. No mutations in plasma samples were detected via SS technique.

### 3.3. Prognostic Value of KRAS Mutations Detected by ARMS–HRMA

After a median follow-up of 12 months (IQR 2–19), 72 (82%) patients had disease progression, and 69 (78%) patients had died.

#### 3.3.1. Tumor Samples

**Figure 3:**
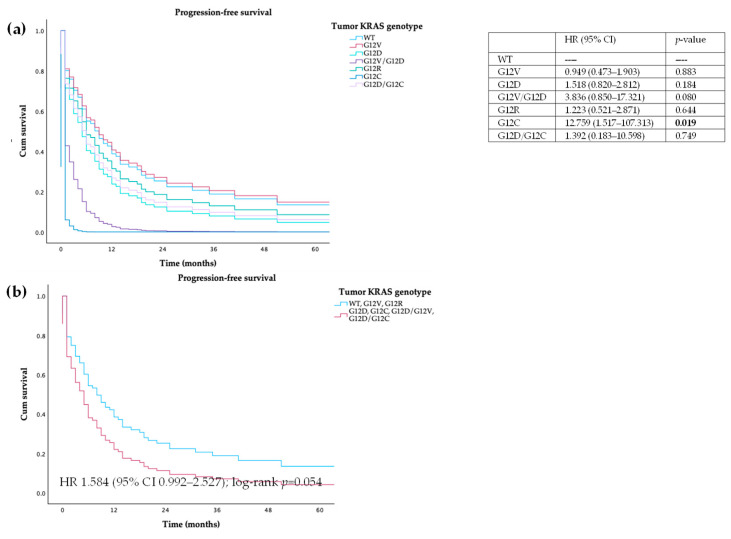
Progression-free survival of patients with PDAC, according to (**a**) *KRAS* mutations and (**b**) *KRAS* category mutations in tumor.

**Figure 4:**
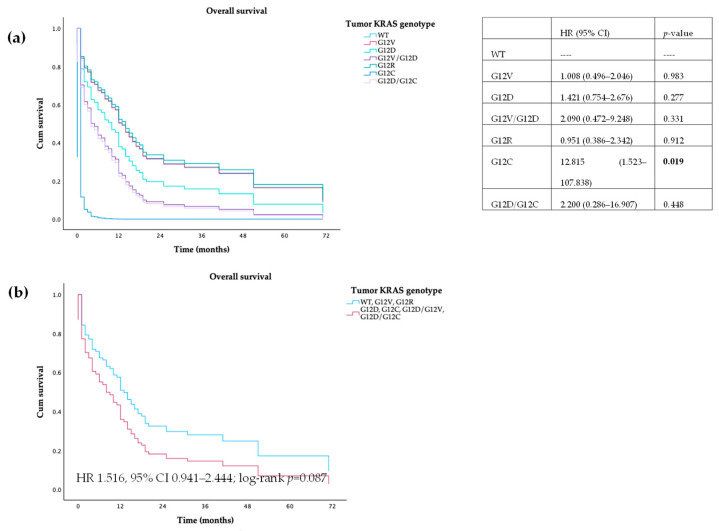
Overall survival of patients with PDAC, according to (**a**) *KRAS* mutations and (**b**) *KRAS* category mutations in tumor.

There was no statistical difference in PFS and OS between patients wild-type or *KRAS*-mutated tumors (HR 1.292, 95% CI 0.739–2.257, log-rank *p* = 0.368; HR 1.234, 95% CI 0.695–2.192, log-rank *p* = 0.473, respectively). When we compared different mutations individually with wild-type patients, differences were found between wild-type and G12C patients, both in PFS (10.0 vs. 0 months, HR 12.759, 95% CI 1.517–107.313; log-rank *p* = 0.019) and OS (12.5 vs. 0 months, HR 12.815, 95% CI 1.523–107.838; log-rank *p* = 0.019). When we grouped patients with WT, G12R, or G12V mutations and compared to patients harboring G12D or G12C tumors, we observed that the latter had a trend towards lower PFS (9 vs. 4 months, HR 1.584, 95% CI 0.992–2.527; log-rank *p* = 0.054) and OS (12 vs. 7 months, HR 1.516, 95% CI 0.941–2.444; log-rank *p* = 0.087)—Figure 3 and Figure 4.

#### 3.3.2. Plasma Samples

**Figure 5:**
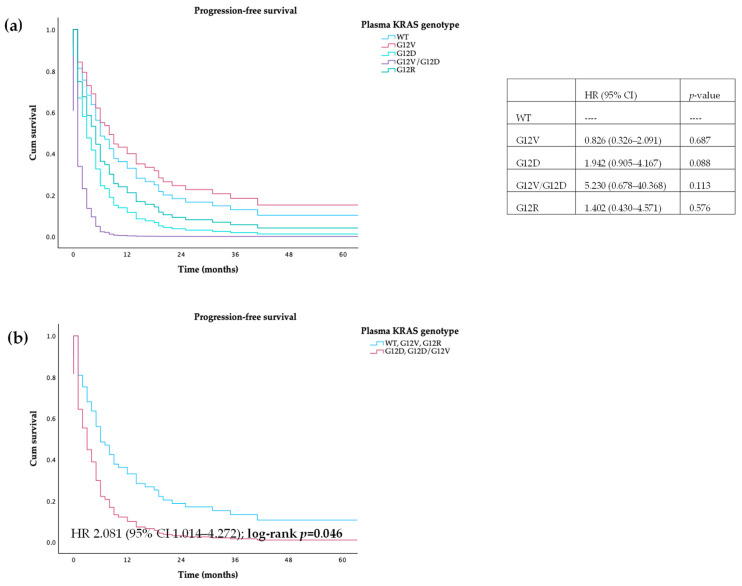
Progression-free survival of patients with PDAC, according to (**a**) *KRAS* mutations and (**b**) *KRAS* category mutations in plasma.

**Figure 6:**
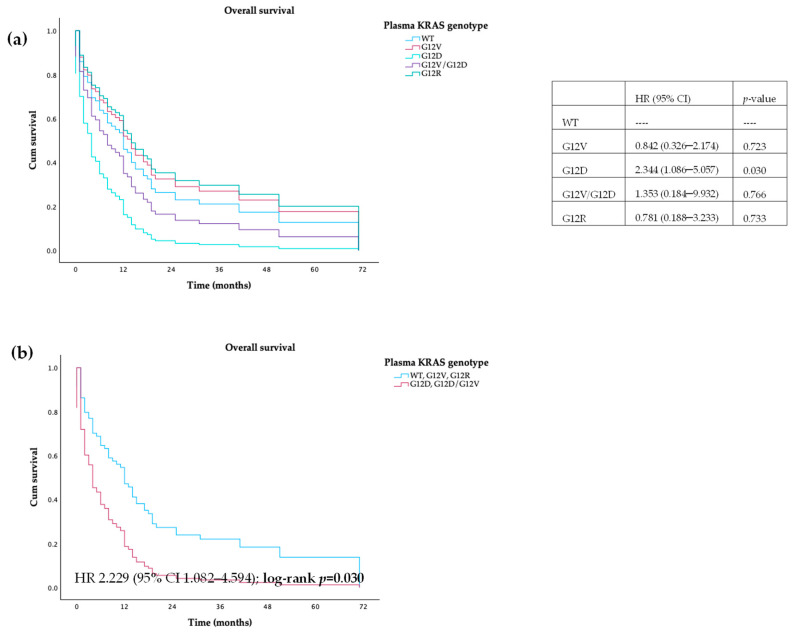
Overall survival of patients with PDAC, according to (**a**) *KRAS* mutations and (**b**) *KRAS* category mutations in plasma.

Regarding stratification of KRAS genotype in plasma, no differences were found between wild-type and different mutations, both in PFS and OS. When we grouped WT and mutations in G12V and G12R and compared to patients harboring G12D mutations, we observed that the latter had a lower PFS (7 vs. 2 months, HR 2.081, 95% CI 1.014–4.272; log-rank *p* = 0.046) and OS (12 vs. 4 months, HR 2.229, 95% CI 1.082–4.594; log-rank *p* = 0.030)—Figure 5 and Figure 6. No differences were found in both PFS and OS in patients with or without mutations detectable in plasma (HR 0.949 (95% CI 0.517–1.742); log-rank *p* = 0.866; HR 0.898 (95% CI 0.490–1.644); log-rank *p* = 0.727, respectively).

No association was found between *KRAS* mutational status and early relapse after surgery (1 year or less) or response to different regimens or chemotherapy, i.e., FOLFIRINOX-like or gemcitabine-based.

### 3.4. Uni and Multivariate Analysis

To minimize the influence of possible confounding factors in the association between *KRAS* mutational category and prognosis, a multivariate analysis was performed with prognostic factors with a *p*-value < 0.100 in univariate analysis for PFS and OS. Uni and multivariate analysis for factors associated with PFS and OS are shown in Table 2 and Table 3, respectively.

In respect to PFS (Table 2), in the univariate analysis, we observed that age, performance status, metastatic disease, and palliative treatment were associated with lower PFS. *KRAS* mutation status in tumor was marginally significant (*p* = 0.054). However, in multivariate analysis, the presence of a G12D and/or G12C mutation in the tumor was the only factor significantly associated with a lower PFS when compared with other *KRAS* status (i.e., WT, G12R and G12V mutations) (HR 1.792, 95% CI 1.061–3.028, *p* = 0.029).

In respect to OS (Table 3), results were similar. In multivariate analysis, we observed that ECOG-PS at diagnosis and tumors with a mutation on G12D and/or G12C were the only factors associated with a lower OS (HR 2.885, 95% CI 1.112–7.487, *p* = 0.029; HR 1.757, 95% CI 1.013–3.049, *p* = 0.045, respectively).

## 4. Discussion

### 4.1. Summary of Main Findings

In this study, we prospectively analyzed a group of 88 patients newly diagnosed with PDAC, applied and validated a method that combines ARMS with HRMA to detect *KRAS* mutations in both tumor and plasma samples, and showed that having a mutation G12D or G12C in tumor or plasma is associated with lower PFS and OS. To our knowledge, this technique has not been used in previous clinical studies and certainly not for clinical decisions in PDAC patients.

### 4.2. Interpretation of Results

Our series includes, predominantly, patients who were treated with upfront surgery (46/88) because we only included patients with collected tumor specimens, and these are easier to collect during surgery. Nonetheless, the remaining 42/88 patients had tumor samples collected during EUS-FNB needed for histologic confirmation before ChT, showing that molecular characterization using EUS-FNB collected specimens is feasible.

We had previously shown that ARMS–HRMA methodology has higher sensitivity and specificity for the detection of mutations in tumor and plasma samples from patients with PDAC than SS [28]. The developed method stands out for its simplicity, costs, and reduced time to results. Herein, we more than doubled the size of our population and still observed the same performance, with SS detecting *KRAS* mutations in 51% of tumor samples (n = 45) and ARMS–HRMA detecting mutations in 77% of tumor samples (n = 68). Additionally, in plasma samples, only ARMS–HRMA could detect *KRAS* mutations. These were detected in 25.4% (n = 18) patients, in full accordance with mutations found in tumors. In the referred previous validation paper by our group [28], using tumor/plasma DNA from 23 patients, ARMS–HRMA methodology was compared to droplet digital PCR (ddPCR) for G12V and G12D mutations. Very similar detection rates were observed between both methodologies, but ARMS–HRMA was able to detect one additional G12D mutation in one of the plasma samples that was not detected by ddPCR. This opens the window for ample use of liquid biopsies in this field using a rapid and cheap technique.

PDAC prognosis remains poor, with only 9% of patients being alive after five years [3]. In addition to late diagnoses in most cases, there has been little progress in developing personalized treatments, leading to uniform therapies that do not account for molecular differences between tumors. Treatment decisions are currently based solely on cTNM staging, even though prior studies have demonstrated the prognostic relevance of *KRAS* mutations, which are an early and almost universal event in pancreatic cancer development. One major reason preventing molecular analysis from being incorporated into treatment decisions in PDAC patients is the complexity and high cost of such analyses, a considerable obstacle to obtaining results quickly enough to influence clinical decisions [27].

In agreement with previous studies [15,16,17,18], we also found that *KRAS* mutations detected in tumor and plasma seem to hold prognostic value, allowing for patients to be split into two categories—better and worse prognosis—according to type of *KRAS* mutations. Patients with tumor samples with G12D or G12C mutations have both a lower PFS (HR 1.792, 95% CI 1.061–3.028, *p* = 0.029) and OS (HR 1.757, 95% CI 1.013–3.049, *p* = 0.045) when adjusted for confounding factors as age, ECOG-PS, clinical stage, and, more importantly, type of treatment. Despite being less sensitive for plasma samples, we also demonstrated that patients with G12D *KRAS* mutations in plasma also had a lower PFS (HR 2.081, 95% CI 1.014–4.272; *p* = 0.046) and OS (HR 1.516, HR 2.229, 95% CI 1.082–4.594; *p* = 0.030). The dismal prognosis of G12D tumors in patients treated with ChT had been shown in previous studies [12,13,14] but in our population, G12D and G12C mutations were also a negative prognostic factor in operated patients, which reinforces its utility in clinical decisions, across all stages of disease.

### 4.3. Comparison with the Existing Literature

We found mutations in codon 12 of KRAS in 77.3% of patients, which is aligned with what has been reported in the literature [30] using more advanced methods like next-generation sequencing (NGS) or ddPCR. However, ARMS–HRMA is significantly quicker (capable of determining *KRAS* mutation status in just six hours), and much more affordable, than NGS, making it easier to apply in clinical practice. In our cohort, *KRAS* mutation frequency distribution is in line with what is described in other studies, where G12D is the most frequent, followed by G12V and G12R [31,32,33].

While sensitivity was much higher for tumor samples, ARMS–HRMA also identified mutations in 25.4% of plasma samples. This is below what has been reported in the literature (ranging from 44% to 67%) [34,35,36,37] using different technologies such as ddPCR or NGS. As stated before, these are more expensive, time-consuming, and not readily available to be used in clinical practice. Recently, Lee et al. [38], using ddPCR for detection of *KRAS* mutations in plasma samples from PDAC patients, detected mutations in 53% of patients. It is worth noting that in this recently published study [29], most patients included (80%) had metastatic disease, which can account for increased amounts of ctDNA [27,37,39]. In our cohort, the majority of samples (83%) are from PDAC resectable/borderline patients, which further highlights the potential of ARMS–HRMA for *KRAS* mutation detection in plasma samples across all PDAC stages. We believe that ARMS–HRMA sensitivity in plasma can be improved, namely sample processing and storage, which are of paramount importance and may be further optimized.

Liquid biopsies are non-invasive tools and allow for longitudinal monitoring of response to treatment [40]. Types of liquid biopsies include ctDNA, exosomes, and circulating tumor cells. Although circulating tumor cells are the most sensitive, they involve complex and costly technology, only available for research purposes. ctDNA, while less sensitive, has proven effective in predicting overall and progression-free survival in PDAC patients [41] and can help to monitor response to treatment, and predict relapse before it becomes visible through imaging or increase in CA19.9 [13,42]. However, given the low levels of ctDNA in plasma, highly sensitive molecular testing is necessary for accurate results [27].

Regarding the prognostic value of different *KRAS* mutations, they are in line with what is described. KRAS G12D mutations have been shown to independently predict worse survival outcomes [12,13,43,44], while G12V mutations may identify tumors with better responses to therapy [45,46]. G12C mutations, although very rare, have also been associated with a worse prognosis [15,16,17,18]. In our study, the prognostic value of these mutation categories was observed both for tumor and plasma samples. These findings are particularly important from our perspective because they may impact treatment decisions. Currently, only a small number of PDAC patients proceed to upfront surgery, and this decision is primarily based on the presence of vascular invasion detected through CT or MRI. Because PDAC is considered by some as a systemic disease from the beginning [11], some authors advocate neo-adjuvant therapy in all patients [5]. However, in a recently published phase 2 trial [47] in patients with resectable tumors, neoadjuvant chemotherapy with modified FOLFIRINOX did not demonstrate a survival benefit compared with upfront surgery. Overall, of patients with borderline resectable tumors who are recommended to receive neo-adjuvant chemotherapy, only 50–60% of these patients are operated on, as the disease progresses in the remainder [5]. Similarly, some patients, despite having resectable tumors, experience early relapse in less than 6 months [48,49]. These scenarios put in evidence the biological heterogeneity of these tumors, which is certainly related to their molecular differences. Given that pancreatic surgery is still a highly invasive procedure with significant morbi-mortality, even in high-volume centers, being able to identify patients with aggressive tumors, more prone to relapse after surgery, could serve as an important factor in surgical decision-making. We may hypothesize that tumors with KRAS G12D and G12C mutations should be treated with medical therapy, chemo, or targeted therapy already available for these specific genotypes, and response to treatment monitored by liquid biopsies. In contrast, less aggressive tumors with WT, G12V, or G12R genotypes could be operated on if no vascular invasion is present on the CT scan. As proposed by Labori et al. [47], future translational research may reveal subgroup differences and novel trials should be biomarker driven.

### 4.4. Strengths and Limitations

This is a prospective bicentric study, with a larger sample size than our previous study and we additionally tested for G12C and G12R mutations, besides the G12D and G12V mutations tested for previously. Our findings provide us with more robust results towards validation and prognostic implication of mutation detection.

However, our study has some limitations. First, the sample size, which needs to be further enlarged. Multicenter studies with larger sample sizes should be conducted to validate our findings, which, for the time being, should be considered as an exploratory trial. Also, we analyzed only four *KRAS* mutations, all in codon 12 of KRAS protein, since other mutations in codons 12, 13, or 61 are very rare. If one considers that this strategy of mutation analysis may become practice changing, it may be easily extended to the remaining mutations, and cost effectiveness assessed since these mutations are extremely rare. Moreover, our analysis focused solely on ctDNA levels at diagnosis, but recent research by Kruger et al. demonstrated that changes in *KRAS*-mutated ctDNA during chemotherapy in 54 advanced PDAC patients were faster and more pronounced than traditional biomarkers. A rapid drop in ctDNA levels indicated an early response to therapy, and repeated measurements during follow-up proved more effective than CA 19.9 in detecting disease progression [34].

Despite these limitations, to the best of our knowledge, this is the first attempt to propose a strategy of personalized treatment in patients with PDAC. With the development of selective KRAS inhibitors, having a sensitive, cost-effective, and quick method for *KRAS* mutations detection may become practice changing. Personalized treatment considering tumor molecular characteristics has been proposed in various other neoplasms, such as lung and colorectal cancer [50,51]. In regard to PDAC, although previous studies show that tumors with G12D mutations have consistently worse prognosis [12,13,14] in contrast to WT tumors, or those carrying G12R or G12V mutations [15,16,17,18], mutation profile is not considered when treating these patients, probably due to the lack of readily available methods. ARMS–HRMA methodology stands out as a very promising and sensitive methodology to quickly detect *KRAS* mutations in all PDAC stages for personalized patient management.

### 4.5. Recommendations for Future Research

A larger, prospective multicenter study with more patients would be necessary to before implementing this strategy in clinical practice. There is also a need to improve the sensitivity of this technique in plasma samples, which would be very useful for a longitudinal follow-up of patients treated with multimodality therapies and highly prone to relapse.

## 5. Conclusions

In summary, the ARMS–HRMA technique appears to be a rapid, accurate, cost-effective, and reliable method for detecting *KRAS* mutations in PDAC tumors and plasma samples. In our cohort, we were able to categorize patients according to *KRAS* mutations in two groups with different prognosis—patients with G12D and G12C mutations had a lower PFS and OS, both for those treated with surgery and/or ChT or just BSC. To the best of our knowledge, this is the first realistic attempt for treating PDAC patients with a personalized, biomarker-driven approach. Future studies should validate our findings, paving the way for personalized therapy in PDAC patients.

## Figures and Tables

**Table 1:** Socio-demographic and clinical baseline characteristics, according to *KRAS* mutational status.

	Total n = 88	*KRAS*-WT n = 20	*KRAS*-Mutated n = 68	*p*-Value
Age at diagnosis (y), median (IQR)	70 (63–75)	71 (67–75)	70 (63–76)	0.870
Gender, n (%)				
Male	50 (57)	16 (80)	34 (50)	0.021
Female	38 (43)	4 (20)	34 (50)	
ECOG-PS at diagnosis *, n (%)				
0 or 1	75 (93)	15 (94)	60 (92)	1.000
(2	6 (7)	1 (6)	5 (8)	
Clinical Stage **, n (%)				
Resectable	49 (57)	8 (44)	41 (60)	0.321
Borderline resectable	10 (12)	4 (22)	6 (9)	
Locally advanced	2 (2)	0	2 (3)	
Metastatic	25 (29)	6 (33)	19 (28)	
Simplified TNM ***, n (%)				
I	19 (22)	6 (30)	13 (20)	0.767
II	22 (26)	4 (20)	18 (28)	
III	15 (18)	3 (15)	12 (19)	
IV	29 (34)	7 (35)	22 (34)	
Treatment, n (%)				
Surgery (chemotherapy)	54 (61)	12 (60)	42 (62)	1.000
Palliative/BSC	34 (39)	8 (40)	26 (38)	
*KRAS* mutations ****, n (%)				
WT	20 (23)	20 (100)	0	----
G12V	22 (25)	0	22 (32)	
G12D	32 (36)	0	32 (47)	
G12R	10 (11)	0	10 (15)	
G12C	1 (1)	0	1 (1)	
G12V/G12D	2 (2)	0	2 (3)	
G12D/G12C	1 (1)	0	1 (1)	

* No data are available in n = 7 patients; ** no data are available in n = 2 patients; *** no data are available in n = 3 patients; **** using ARMS–HRMA. BSC—best supportive care; ECOG-PS—Eastern Cooperative Oncology Group Performance Status; WT—wild type; y—years.

**Table 2:** Factors associated with progression-free survival (univariate and multivariate Cox regression analysis).

	Univariate Analysis			Multivariate Analysis		
	HR	CI 95%	*p*-Value	HR	CI 95%	*p*-Value
Age at diagnosis (y)	1.034	1.002–1.066	0.035	1.020	0.987–1.054	0.247
Gender						
Female	-	-	-	-	-	-
Male	1.167	0.731–1.861	0.517	-	-	-
ECOG-PS at diagnosis						
0 or 1	-	-	-	-	-	-
≥2	2.872	1.204–6.849	0.017	2.885	0.871–5.547	0.096
Clinical Stage						
Non metastatic	-	-	-	-	-	-
Metastatic	1.970	1.187–3.270	0.009	1.632	0.724–3.682	0.238
Treatment						
Surgery ± chemotherapy	0.505	0.311–0.820	0.006	0.685	0.307–1.529	0.355
Palliative/BSC	-	-	-	-	-	-
KRAS mutations in plasma						
No	-	-	-	-	-	-
Yes	0.898	0.490–1.644	0.727	-	-	-
Tumor KRAS status						
WT, G12V, G12R	-	-	-	-	-	-
G12D, G12C, G12D/G12V, G12D/G12C	1.584	0.992–2.527	0.054	1.792	1.061–3.028	0.029

BSC—best supportive care; CI—confidence interval; ECOG-PS—Eastern Cooperative Oncology Group Performance Status; HR—hazard ratio; WT—wild type; y—years.

**Table 3:** Factors associated with overall survival (univariate and multivariate Cox regression analysis).

	Univariate Analysis			Multivariate Analysis		
	HR	CI 95%	*p*-Value	HR	CI 95%	*p*-Value
Age at diagnosis (y)	1.043	1.011–1.077	0.009	1.020	0.987–1.054	0.247
Gender						
Female	-	-	-	-	-	-
Male	1.308	0.808–2.120	0.275	-	-	-
ECOG-PS at diagnosis						
0 or 1	-	-	-	-	-	-
≥2	4.021	1.648–9.812	0.002	2.198	1.112–7.487	0.029
Clinical Stage						
Non metastatic	-	-	-	-	-	-
Metastatic	2.204	1.130–3.709	0.003	1.689	0.707–4.038	0.238
Treatment						
Surgery ± chemotherapy	0.434	0.264–0.714	0.001	0.550	0.234–1.290	0.169
Palliative/BSC	-	-	-	-	-	-
KRAS mutations in plasma						
No	-	-	-	-	-	-
Yes	0.949	0.517–1.742	0.866	-	-	-
Tumor KRAS status						
WT, G12V, G12R	-	-	-	-	-	-
G12D, G12C, G12D/G12V, G12D/G12C	1.516	0.941–2.444	0.087	1.757	1.013–3.049	0.045

BSC—best supportive care; CI—confidence interval; ECOG-PS—Eastern Cooperative Oncology Group Performance Status; HR—hazard ratio; WT—wild type; y—years.

## Data Availability

The original contributions presented in the study are included in the article, further inquiries can be directed to the corresponding author.
